# Are lifestyle factors significantly associated with self-rated health among Japanese female healthcare students?

**DOI:** 10.1186/s12889-021-10435-2

**Published:** 2021-03-15

**Authors:** Makoto Ohtsuki, Yusuke Wakasugi, Takuhiro Narukawa, Shunsuke Uehara, Takeshi Ohkubo

**Affiliations:** 1 Department of Clinical Nutrition, Faculty of Health Science, Suzuka University of Medical Science, 1001-1 Kishioka-cho, Suzuka, Suzuka City, Mie Japan; 2 Department of Medical Welfare, Faculty of Health Science, Suzuka University of Medical Science, 1001-1 Kishioka-cho, Suzuka City, Mie Japan; 3 Department of Health and Nutrition, Sendai Shirayuri Women’s College, 6-1 Honda-cho, Izumiku, Sendai, Japan

**Keywords:** Cross-sectional study, Japanese female healthcare students, Lifestyle, Physique recognition, Self-rated health

## Abstract

**Background:**

Self-rated health (SRH), a subjective perception of an individual’s overall health, is widely used in public health assessment. The transition from adolescence to young adulthood is a critical period involving substantial changes in lifestyle and dietary habits. Therefore, it is important to understand SRH among young Japanese females. The present study aimed to investigate the relationships between SRH among female healthcare students and their lifestyle factors, such as living status (living with others or living alone), smoking habit, alcohol consumption, frequency of breakfast consumption (FBC), physical activity, insomnia, and physique recognition.

**Methods:**

A cross-sectional survey was conducted on 1101 female healthcare students in Japan. The body mass index was calculated from the body height and weight using data from periodic health examinations. Self-reported sociodemographic, lifestyle or dietary habits, physical activity, and SRH were used through a self-administered questionnaire. Participants were classified as having either good SRH (excellent, very good, or good) or impaired SRH (fair or poor). Multivariate logistic regression analysis was performed to investigate the independent relationships between SRH and lifestyle factors.

**Results:**

A total of 11.4% participants demonstrated impaired SRH. Multivariate logistic regression analysis showed that the University of California Los Angeles activity score, Athens Insomnia Scale, and physique recognition were associated with SRH.

**Conclusions:**

It was suggested that public health activities that consider physical activity, sleep, and physique recognition may help maintain and improve SRH in female university students in Japan.

## Background

Self-rated health (SRH), a subjective perception of an individual’s overall health, is a widely used measure in public health assessment. Typically, SRH is examined using a single question, e.g., asking respondents to rate their overall health on a scale from excellent to poor [1]. According to Kaplan, individuals with better SRH exhibit higher survival rates compared with those with poorer SRH, regardless of the presence or absence of disease [2]. Additionally, SRH affects life expectancy, an index that is highly predictive of mortality risk [2]. Furthermore, the validity of SRH measures to predict mortality risk has previously been established in multiple studies [3, 4]. Study results indicate that SRH can be affected by several lifestyle, diet, and biochemical factors [5, 6].

SRH of the general population in Japan is officially assessed every 3 years via the Comprehensive Survey of Living Conditions (CSLC), a nationwide cross-sectional survey of a representative sample of the country’s general population. The CSLC findings from 2016 found that 86.7% of males and 84.4% of females reported excellent, very good, or good SRH [7].

SRH assessment among adolescents is an effective way to evaluate their self-concept of health [8, 9]. In adolescents, a high SRH score is strongly associated with general well-being and psychosomatic symptoms [10, 11]. In Japan, studies have examined whether the younger generation’s knowledge affects their health behaviors or SRH. For example, one study examined whether an accurate knowledge about the healthiness of foods contributed to desirable eating behaviors among of female university students [12]. Another study examined smoking knowledge and practices among male university students. It has been confirmed that a correlation is present between the awareness of bad lifestyle habits and smoking behaviors, healthy eating habits, and personality traits (e.g., feeling inferior) [13].

The transition from adolescence to young adulthood is a critical period during which an individual’s pre-existing habits may be disrupted [14]. Leaving one’s parent’s home and handling the education system at this age are both associated with negative changes in diet [15], which can have significant and long-lasting effects on the future health of individuals and their families [16, 17].

According to an international survey of university students from 22 countries, 70% of Japanese females reported that they were attempting to lose weight [18]. Moreover, Japanese females reportedly exhibit a strong desire to have a thin physique [19]. The annual National Health and Nutrition Survey conducted by the Ministry of Health, Labour and Welfare suggests that young Japanese females tend to have a thin physique. The 2016 survey found that there was a significant increase in the number of females who were “thinner (low body weight)” with a body mass index (BMI) of < 18.5 compared with the proportion 10 years earlier, with 20.7% of women in their 20s falling into this category. Being thin is associated with several health issues in young women, and malnutrition among young women and pregnant women may increase the risk of lifestyle diseases in their children [20–23]. Therefore, it is important to examine and understand the relationships between the lifestyle and SRH among young Japanese females.

The aim of the present study was to investigate the relationships between SRH among Japanese female students and their lifestyle factors, such as living status, smoking habit, alcohol consumption, frequency of breakfast consumption (FBC), physical activity, insomnia, and physique recognition.

## Methods

A cross-sectional study was conducted among 1305 female students who participated in a periodic health examination between April and May 2018 at a Japanese medical university. The students were from nine departments: radiological technology, clinical nutrition, physiotherapy, medical welfare, acupuncture, clinical engineering, medical information science, pharmaceutical sciences, and nursing. All participants provided written informed consent before participating in the study and were informed that there was no penalty for choosing not to participate. The paper-based questionnaire was distributed only to students who had provided written consent. Students who received the questionnaire completed it during the physical examination of their periodic health examination.

Body height and weight data were obtained from the periodic health examination. BMI was calculated as weight in kg/height in m^2^. Global SRH measures typically include the question “How would you rate your overall health?” with five response categories ranging from excellent to poor (i.e., excellent, very good, good, fair, and poor). These five categories are dichotomized into either good health (excellent, very good, or good) or impaired health (fair or poor) [24–26].

The seven independent variables for this study were grade level (1–2 or ≥ 3), living status (living with others or living alone), smoking habit (none, past smoker, or current smoker), alcohol consumption (none, a few times per month, a few times per week, or daily), University of California Los Angeles (UCLA) activity score (< 5 points or ≥ 5 points) [27], Athens Insomnia Scale (AIS) (no insomnia or insomnia) [28–30], and FBC (< 6 days/week or ≥ 6 days/week). We classified the participants into three groups based on physique recognition (“Want to be underweight,” “Want to be overweight,” or “Want to be normal weight”), as measured by the relationship between their current BMI and their desire to lose or gain weight.

Physical activity was assessed using the UCLA activity score [27, 28], a simple scale that ranges from 1 to 10. Participants indicated their most appropriate activity level with 1 to indicate “no physical activity, dependent on others” and 10 to indicate “regular participation in impact sports.”

AIS is a self-assessment insomnia scale created by the World Health Organization for the World Project on Sleep and Health. This instrument’s reliability and validity have already been verified in other studies [29–31]. AIS items measure waking during the night, early morning awakening, total sleep duration, sleep quality, and daytime sleepiness. Each of the scale’s eight questions is answered from 0 (no problem) to 3 (a very serious problem). A total score of ≥4 on the selected items shows suspected insomnia, and a total score of ≥6 indicates insomnia.

**Fig. 1:**
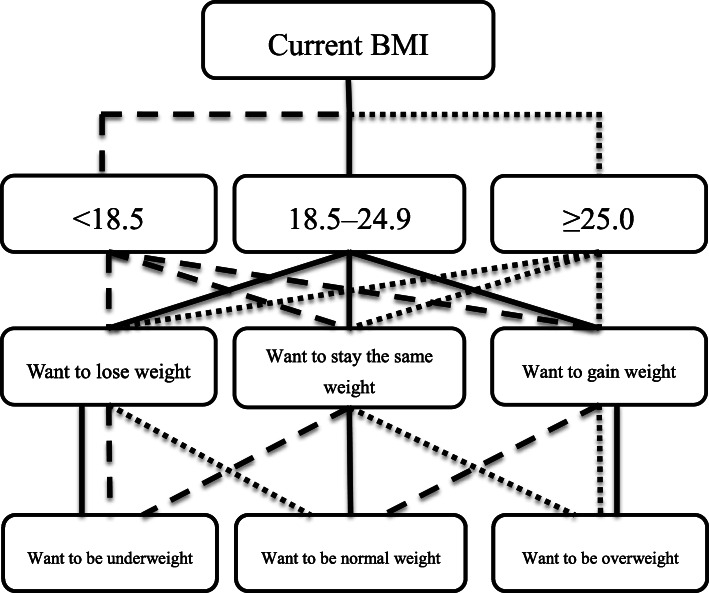
Classification of physique recognition. The solid line represents ideal physique from BMI 18.5–24.9, the small dotted line indicates ideal physique from BMI ≥25.0 and the large dotted line indicates ideal physique from BMI < 18.5. BMI: Body mass index

In recent years, some studies have investigated body image among university students; the Health Behaviour in School-Aged Children (HBSC) study is one such example [18, 32, 33]. However, in these studies, although the self-reported BMI was used to determine an individual’s body image, the self-reported height and weight bias was cited as a study limitation. Therefore, in this study, based on their current BMI, which was obtained via physical examination, we asked the participants “Do you want to gain or lose weight in the future”? We classified their responses into three categories: “I want to lose weight,” “I want to stay the same weight,” and “I want to gain weight” (Table 1). Furthermore, based on Table 1, we defined physique recognition into the following three groups: “Want to be underweight,” “Want to be overweight,” and “Want to be standard weight.” If their current BMI was < 18.5 but they desired to lose weight or stay the same weight, participants were defined as wanting to be underweight. Those with a current BMI < 18.5 who desired to gain weight were defined as wanting to achieve standard weight. Those with a current BMI of ≥25.0 who desired to gain weight or stay the same weight were defined as wanting to be overweight, and if they desired to lose weight, they were defined as wanting to achieve standard weight. If their current BMI was 18.5–24.9, participants who desired to lose weight were classified as the wanting to be underweight group, those who desired to gain weight were classified as the wanting to be overweight group, and those who desired to stay the same weight were classified as the wanting to remain at the standard weight group (Fig. 1).

**Table 1:** Do you want gain or lose weight in the future from your current level?

BMI (kg/m^2^)	All	I want to lose weight	I want to stay the same weight	I want to gain weight	*p*-value^*^
< 18.5	207 (18.8)	79 (38.2)	97 (46.9)	31 (14.9)	
18.5–24.9	805 (73.1)	713 (88.6)	88 (10.9)	4 (0.5)	
≥25.0	89 (8.1)	88 (98.9)	1 (1.1)	0 (0.0)	< 0.0001

Data are expressed as n (%). ^*^Chi-square test was used. *BMI* body mass index

Participants were classified as having either good SRH (excellent, very good, or good) or impaired SRH (fair or poor). Differences in grade level, living status, smoking habit, alcohol consumption, UCLA activity score, AIS score, FBC, and physique recognition were compared using the Chi-square test, as appropriate.

Univariate logistic regression analysis was used to compute the odds ratios and 95% confidence interval. *P*-value < 0.05 indicated statistical significance. In addition, multiple logistic regression analysis was performed to adjust for these factors. All statistical analyses were conducted using JMP 9.0.2 (SAS Institute Inc., Cary, NC, USA).

## Results

Overall, 1305 female students participated in the periodic health examination, of which 1101 completed the questionnaire, thereby resulting in a response rate of 84.4%. Mean participant age was 19.7 years [standard deviation (SD) = 2.7; range = 18–46 years], and mean BMI was 20.9 (SD = 3.0; range = 14.3–38.4).

Table 1 shows the responses to the question “Do you want to gain or lose weight in the future?” based on their current BMI data, which were classified as physique recognition. Several students with a BMI of < 18.5 or 18.5–24.9 desired to lose weight. The distribution of participant characteristics based on whether they had good or impaired SRH is shown in Table 2. For physique recognition, the participants who desired to lose weight tended to believe that they were healthy. Table 3 shows the results of the logistic regression analysis of factors associated with impaired SRH in all participants. For the univariate analysis, although grade ≥ 3, FBC for < 6 days/week, smoking history (past smoker), UCLA activity score < 5 points, and AIS score indicative of insomnia were positively correlated with impaired SRH, a negative correlation was observed with the wanting to be underweight group. Likewise, in the multivariate analysis, although the UCLA activity score < 5 points, and AIS score indicative of insomnia were positively correlated with impaired SRH, there was a negative correlation with the wanting to be underweight group.

**Table 2:** Characteristics of the groups with good and poor/fair SRH

	Total(*n* = 1101)	Good SRH (*n* = 975)	Poor/fair SRH (*n* = 126)	*p*-value^*^
	n (%)	n (%)	n (%)	
Grade				
1–2	617 (56.0)	558 (90.4)	59 (9.6)	
≥ 3	484 (44.0)	417 (86.1)	67 (13.9)	0.027
Living status				
Living with others	812 (73.8)	726 (89.4)	86 (10.6)	
Living alone	289 (26.2)	249 (86.1)	40 (13.9)	0.136
FBC				
< 6 days/week	367 (33.3)	310 (84.5)	57 (15.5)	
≥ 6 days/week	734 (66.7)	665 (90.6)	69 (9.4)	0.003
Smoking				
None	1083 (98.4)	964 (89.0)	119 (11.0)	
Past smoker	12 (1.1)	7 (58.3)	5 (41.7)	
Current smoker	6 (0.5)	4 (60.0)	2 (40.0)	0.001
Alcohol drinking				
None	820 (74.5)	731 (89.1)	89 (10.9)	
A few times/month	182 (16.5)	160 (87.9)	22 (12.1)	
A few times/week	91 (8.3)	78 (85.7)	13 (14.3)	
Daily	8 (0.7)	6 (71.4)	2 (28.6)	0.470
UCLA activity score				
≥ 5 points	285 (25.9)	265 (93.0)	20 (7.0)	
< 5 points	816 (74.1)	710 (87.0)	106 (13.0)	0.006
AIS scores				
Non-insomnia group	872 (79.2)	813 (93.2)	59 (6.8)	
Insomnia group	229 (20.8)	162 (70.7)	67 (29.3)	< 0.0001
Physique recognition				
Want to be underweight	889 (80.7)	805 (90.6)	84 (9.4)	
Want to be normal weight	207 (18.8)	167 (80.7)	40 (19.3)	
Want to be overweight	5 (0.5)	3 (60.0)	2 (40.0)	< 0.0001

^*^Chi-square test was used. *FBC* frequency of breakfast consumption, *UCLA* University of California Los Angeles, *AIS* Athens Insomnia Scale, *SRH* self-rated health

**Table 3:** Uni- and multivariate analyses of factors associated with poor/fair SRH

**Univariate analysis**	**Odds Ratio**	**95% CI**	***p*-value**^**§**^
Grade			
1–2	Reference		
≥ 3	1.52	1.04–2.20	0.028
Living status			
Living with others	Reference		
Living alone	1.35	0.91–2.03	0.137
FBC			
≥ 6 days/week	Reference		
< 6 days/week	1.77	1.22–2.88	0.003
Smoking			
None	Reference		
Past smoker	5.79	1.81–18.52	0.003
Current smoker	4.05	0.73–22.40	0.109
Alcohol drinking			
None	Reference		
A few times/month	1.13	0.69–1.89	0.631
A few times/week	1.37	0.73–2.56	0.326
Daily	2.73	0.54–13.77	0.222
UCLA activity score			
≥ 5 points	Reference		
< 5 points	1.98	1.20–3.26	0.007
AIS			
Non-insomnia group	Reference		
Insomnia group	5.70	3.86–8.40	< 0.0001
Physique recognition			
Want to be normal weight	Reference		
Want to be overweight	2.78	0.45–17.21	0.271
Want to be underweight	0.44	0.29–0.66	< 0.0001
**Multivariate analysis**			
^a^ Grade			
1–2	Reference		
≥ 3	1.31	0.88–1.97	0.184
^b^ FBC			
≥ 6 days/week	Reference		
< 6 days/week	1.32	0.87–2.00	0.191
^c^ Smoking			
None	Reference		
Past smoker	2.33	0.63–8.64	0.206
Current smoker	2.52	0.42–14.97	0.310
^d^ UCLA activity score			
≥ 5 points	Reference		
< 5 points	1.87	1.11–3.16	0.019
^e^ AIS			
Non-insomnia group	Reference		
Insomnia group	5.38	3.59–8.06	< 0.0001
^f^ Physique recognition			
Want to be normal weight	Reference		
Want to be overweight	3.15	0.47–21.14	0.238
Want to be underweight	0.44	0.28–0.68	0.000

^§^Logistic regression analysis was used. *FBC* frequency of breakfast consumption, *UCLA* University of California Los Angeles, *AIS* Athens Insomnia Scale, *SRH* self-rated health

^a^Multivariable model was adjusted for FBC, smoking, UCLA activity score, AIS, and physique recognition

^b^Multivariable model was adjusted for grade, smoking, UCLA activity score, AIS, and physique recognition

^c^Multivariable model was adjusted for FBC, grade, UCLA activity score, AIS, and physique recognition

^d^Multivariable model was adjusted for FBC, grade, smoking, AIS, and physique recognition

^e^Multivariable model was adjusted for FBC, grade, smoking, UCLA activity score, and physique recognition

^f^Multivariable model was adjusted for FBC, grade, smoking, UCLA activity score, and AIS

## Discussion

The present study examined the relationships between SRH and lifestyle habits of Japanese female healthcare students. The results indicated that the UCLA activity score, AIS score, and physique recognition were significantly associated with impaired SRH. Overall, this cross-sectional observational study of lifestyle habits among Japanese female healthcare students showed that lower physical activity and insomnia were significantly positively associated with impaired SRH. Further, the desire to be underweight showed a significant negative relationship with impaired SRH.

Insomnia has been identified as the most common sleep disorder [34]. Various risk factors for insomnia, including female sex and obesity—both of which increase the risk of chronic insomnia, have been identified in the general population [35]. In the present study sample of female university students, we observed low levels of obesity but high levels of insomnia. A cross-sectional study of students from seven universities in Poland found a strong positive correlation between the levels of insomnia and stress [36]. Another study in Japan validated the simplified Japanese version of the AIS (AIS-SJ) in relation to psychological issues [37]. Therefore, the students in our study may have had impaired SRH because of stress or psychological issues.

The UCLA activity score is used to evaluate the physical activity of patients with joint replacement [27]. SRH was positively associated with physical activity in the present study, although not many achievements as physical activity exists for evaluating healthy people [28]. Therefore, the validity or reliability of the testing of this scale has not been established. However, this result is consistent with other findings regarding the relationship between physical activity and SRH in young adults [38, 39]. Several possible hypotheses could explain the relationship between physical activity and health, including hypotheses from physiological [40] and psychological viewpoints [41]. However, because females tend to judge their SRH as good or bad based on various factors (e.g., pain and mental/physical symptoms [42]), these findings likely reflect several combinations of factors. Regardless of the precise reason, an independent association is present between a lower physical activity level and impaired SRH, which necessitates public health programs to encourage regular physical activity.

Most study participants were either normal weight or underweight, with very few being overweight. However, majority of them wished to have a thinner physique. It is noteworthy that the group who desired to be underweight still considered itself to be healthy. Baba et al. reported that the desire to be thinner was positively correlated with a sense of gain or loss about one’s body, being praised, and sex role acceptance; however, it was negatively correlated with self-esteem and stress [43]. These psychological factors might affect the desire to be thin because individuals may feel a sense of merit in losing weight. This tendency has been observed since elementary school, and it is believed that exposure to media that stimulates fashion consciousness as well as the ideal way of the fashion industry being lean-oriented are background factors for these beliefs [44]. These perceptions pose a risk to the health of young women who are already in the normal weight range and do not need to lose weight to be healthy. In fact, the estimated daily energy requirement for Japanese women in their 20s is 2000 kcal [45], while the actual average energy intake is 1643 kcal [46]. Since 2002, the food intake has been low at approximately 1600 kcal. This is less than the estimated energy requirement of 1700 kcal for Japanese girls aged 8–9 years [45]. One study conducted on young Japanese women [47] reported that groups of underweight females—both those with and without a desire to be thinner—required nutritional education to help them maintain an appropriate body weight. Thus, we suggest that any educational intervention designed to help young females understand and correctly rate their own health and understand their appropriate body weight should include a psychological approach along with nutritional and health education. In addition, if they believe that being thin and fashionable makes them beautiful and healthy, we need to conduct public health activities that correct the perception among women and change societal norms regarding healthy weight and the importance of eating adequate and healthy food in women.

This study had some limitations. First, it was conducted in a medical university and was thus not population based. These participants may have followed healthier lifestyles and been more health-conscious than the general population of the same age. Therefore, caution should be exercised when generalizing these results to the general population, even among those in the same age group. Although the study’s convenience sampling may have contributed to selection bias, the students showed high levels of incorrect physique recognition, suggesting that the results are likely to be valid. Second, in the present study, we asked participants if they would like to gain weight from the BMI actually obtained by physical examination and further classified each student’s ideal body physique by BMI and their self-report. However, this survey method is a new approach that incorporates the survey methods of Noh et al. [48] with reference to the HBSC survey method; therefore, further empirical testing is required.

Third, this study was conducted using a self-administered questionnaire; therefore, there may be a recall bias, and our perception of subjective health may be different. It was unclear whether subjects had low SRH because they had symptoms and illness despite being satisfied with their body shape or had higher SRH because they had no symptoms or illness despite being dissatisfied with their body shape in this study. In the future, it is a more detailed examination based on the concept of health is warranted. Fourth, in the present survey, participants were asked to complete a questionnaire about their desired body physique; this might lead to a social desirability bias, which projects a favorable image of themselves to avoid receiving a negative evaluation. Finally, because this was a cross-sectional study, causal relationships could not be determined. In the future, investigation is warranted to ascertain whether physical activity, sleeping, and physique recognition are the results or causes of SRH.

## Conclusion

We conducted a cross-sectional study to investigate the relationships between SRH and lifestyle among female healthcare students at a Japanese medical university. The UCLA activity score, AIS score, and physique recognition were all significantly associated with impaired SRH. Therefore, it was suggested that public health activities that consider physical activity, sleep, and physique recognition may help maintain and improve SRH of female university students in Japan.

## Data Availability

The datasets used and/or analyzed during the current study are available from the corresponding author on reasonable request.

## Abbreviations

AIS: Athens Insomnia Scale
BMI: Body mass index
CSLC: the Comprehensive Survey of Living Conditions
FBC: Frequency of breakfast consumption
HBSC: Health Behavior in School-Aged Children
SRH: Self-rated health
UCLA: University of California Los Angeles
